# Scientific Items

**Published:** 1882-06-20

**Authors:** 


### SCIENTIFIC ITEMS.

**The Telephone** is now made to serve the diver in reporting what he has discovered or in receiving instructions from above. One of the glasses made in the helmet is replaced by a sheet of copper, into which the telephone is fixed, so that the diver, when at the bottom of the sea, has only to turn his head slightly in order to receive directions. It was the custom formerly to pull the diver up at intervals for reports and instructions, which was more or less dangerous.—*Mechanical News.*

**How Fast Does Niagara Recede?**—The river distance from the falls to the mouth of the canyon is about ten miles. If the maximum estimate of our engineers of an inch per year is allowed, the falls have been 1,267,200 years in reaching their present position. The river distance to Buffalo is 2½ times as long, an equivalent of 3,168,000 years. The bed of the river from the falls to the outlet of Lake Erie was ascertained by thousands of soundings to be bed-rock, so that a natural drainage of the great lakes by the Niagara river need not be anticipated as long as man may be permitted to exist.—*Mechanical News.*

**Unexplored Countries.**—According to an English geographical writer, there are four vast areas still to be opened up or traversed by civilized man, and which, among them, constitute about one-sixteenth of the whole area of the globe. Of these, there is the ant-arctic region, which in extent is about seventy-five times that of Great Britain; the second lies about the north pole; the third is Central Africa, and the fourth is Western Australia. The south polar region referred to is almost conterminous with the antarctic circle. The vast African area reaches on the west very closely to the coast, and it is only near the equator that it has more than superficially been driven inland. In Australia, the great undeveloped region is that which lies west of the track explored from North to South by Stuart, and which now forms the line of telegraphic communication across that continent.—*Mechanical News.*

**Effect of Compression on Solids.**—A German chemist has recently published an interesting memoir, giving the results of a series of experiments as to the effect of powerful compression on various bodies. The substances experimented with were taken in fine powder, and submitted in a steel mould to pressures varying from 2000 to 7000 atmospheres, or about 7000 kilograms per square centimeter. Lead filings at a pressure of 2000 atmospheres, were transformed into a solid block, which no longer showed the least grain under the microscope, and the density of which was 11.5, while that of ordinary lead is 11.3 only. At 5000 atmospheres the lead became fluid and ran out through all the interstices of the apparatus. The powders of zinc and bismuth, at 5000 to 6000 atmospheres, gave solid blocks having a crystaline fracture. To ward 6000 atmospheres zinc and tin appeared to liquify. Powder of prismatic sulphur was transformed into a solid block of octahedric sulphur. Soft sulphur and octahedric sulphur led to the same result as prismatic. Red phosphorus appeared also to pass into the denser state of block phosphorus. A certain number of pulverized salts solidify through pressure, and become transparent, thus proving the union of the molecules. At high pressure the hydrated salts, such as sulphate of soda, can be completely liquefied. Various organic substances, such as fatty acids, damp cotton and starch, change their appearance, lose their texture, and consequently undergo considerable molecular change.—*Boston Journal of Chemistry.*

**M. Pasteur.**—It required no ordinary moral courage for M. Pasteur, the distinguished biologist and investigator in the department of germinal organisms, to announce himself a spiritualist, at his brilliant reception at the French Academy, April 27th. His position was perhaps as enviable as any that a scientific man can hope for, surrounded as he was by an assemblage which embraced the most learned and brilliant men of the age. It was the occasion of his introduction to the Academy, when he gave an address commemorative of M. Littre, to whose chair he succeeds.

It is not supposable that, in the use of the term, he intended to have his associates understand that he has any affiliations with the long-haired, peripatetic philosophers who exhibit as mediums in various parts of the world, but rather that he discovers in the psychical phenomena called spiritualistic the elements of fact. A more extended, careful, unbiassed study of this class of phenomena by scientific men has undoubtedly resulted in establishing in the minds of many a clear conviction of their verity; but there are few who have the courage openly to avow their belief.—*Boston Journal of Chemistry.*

IN the tropics of the Old World the annual rainfall is, according to Dana, about 77 inches, while it is 155 inches in South America. In the Eastern United States, it is 40 to 50 inches; but west of the one-hundredth meridian, beyond the Mississippi to the Sierra Nevada, it is mostly 12 to 16 inches. The annual amount in Great Britain averages 35 inches; in France, 20 to 11 inches; farther from the coast, in Central Germany and Russia, only 15 to 20 inches; but about the Alps, it is mostly 35 to 50 inches.—*Ex.*

THE degree of heat necessary to destroy trichinæ in pork is a matter of importance as a safeguard against trichinosis. A German microscopist states that only the most thorough cooking of meat will insure perfect safety, as pork cooked to the degree known as "rare" may still contain the living parasites so much dreaded.—*Ex.*

THE researches of P. Plantamour have shown that every rise in temperature is accompanied by an elevation of the ground, and a fall of the thermometer is marked by a sinking of the ground level. The extent of the movements is in some cases quite remarkable—*Ex.*

